# Carbapenem-Resistant *Klebsiella pneumoniae* in Healthcare-Associated Infections: Global and Regional Epidemiology, Resistance Mechanisms, and Therapeutic Strategies, with Particular Attention to Romania and Eastern Europe (2020–2025)

**DOI:** 10.3390/microorganisms14091870

**Published:** 2026-08-23

**Authors:** Oana-Elena Ioniţă, Roxana-Carmen Cernat, Nicola-Maria Militaru, Maria-Elena Vodarici, Maria Fulina, Daniela Pițigoi, Elena Mocanu, Beatrice Severin, Claudia-Simona Cambrea, Irina-Magdalena Dumitru

**Affiliations:** 1 Department of Infectious Diseases and Epidemiology, Faculty of Medicine, Ovidius University of Constanta, 1 University Alley, 900470 Constanta, Romania; wanamed@ymail.com (O.-E.I.); mariaelenavodarici@yahoo.com (M.-E.V.); cambrea.claudia@gmail.com (C.-S.C.);; 2 Doctoral School, Faculty of Medicine, Ovidius University of Constanta, 1 University Alley, 900470 Constanta, Romania; 3 Clinical Infectious Diseases Hospital, Ferdinand Blvd. No. 100, 900709 Constanta, Romania; 4 County Clinical Emergency Hospital “St. Apostle Andrew”, Tomis Blvd. No. 145, 900591 Constanta, Romania; maria.fulina@yahoo.com; 5 Department of Epidemiology I, University of Medicine and Pharmacy “Carol Davila”, Bd. Eroii Sanitari 8, Sector 5, 050474 Bucharest, Romania; daniela.pitigoi@umfcd.ro; 6 National Institute of Infectious Diseases “Prof. Dr. Matei Balș”, 1 Dr. Calistrat Grozovici Street, District 2, 021105 Bucharest, Romania; 7 Department of Public Health and Management, Faculty of Medicine, Ovidius University of Constanta, 1 University Alley, 900470 Constanta, Romania; drmocanu@yahoo.com; 8 Department of Hygiene, Faculty of Medicine, Ovidius University of Constanta, 1 University Alley, 900470 Constanta, Romania; 9 Academy of Romanian Scientists, 3 Ilfov Street, 050044 Bucharest, Romania

**Keywords:** *Klebsiella pneumoniae*, carbapenem resistance, healthcare-associated infections, molecular epidemiology, clinical outcomes, antimicrobial stewardship

## Abstract

Healthcare-associated infections (HAIs) caused by multidrug-resistant *Klebsiella* spp. represent a critical and escalating global public health threat. Carbapenem-resistant *Klebsiella pneumoniae* (CRKP) has been designated a critical-priority pathogen by the World Health Organization, and in the 2024 WHO Bacterial Priority Pathogens List, it was the top-ranked pathogen overall. The convergence of carbapenem resistance with hypervirulence in emerging strains has further complicated therapeutic decision-making. This review provides a narrative synthesis of the evidence published between 2020 and 2025 on the prevalence, resistance mechanisms, molecular epidemiology, clinical outcomes, and therapeutic strategies for *Klebsiella pneumoniae* infections acquired in healthcare settings, with particular attention to the Eastern European and Romanian context. PubMed/MEDLINE, Embase, Web of Science, and the Cochrane Library were searched for relevant publications from January 2020 to June 2025, supplemented by WHO and ECDC surveillance reports. Studies were selected narratively for their relevance to the themes addressed. No new quantitative pooling was undertaken; all summary estimates reported below are cited from the published meta-analyses and surveillance reports that generated them. In the most recent global meta-analysis of hospital-acquired CRKP infection, which pooled 61 studies and 513,307 patients from 14 countries, the global prevalence of CRKP among nosocomial *K. pneumoniae* infections was 28.69% (95% CI: 26.53–30.86%), with pronounced regional variation from 14.29% in high-income North America to 66.04% in South Asia, and 42.05% in Western Europe. Pooled mortality among patients infected with CRKP has been estimated in a separate meta-analysis at 42.14%, compared with 21.16% among patients infected with carbapenem-susceptible strains, rising to 54.30% in bloodstream infections. Surveillance data place Romania third in Europe for carbapenem resistance among invasive *K. pneumoniae* isolates, at 50.30%, with a distinctive predominance of NDM plus OXA-48-like co-producers. Ceftazidime-avibactam is recommended for KPC- and OXA-48-producing strains, whereas metallo-beta-lactamase producers require aztreonam-containing combinations. CRKP in HAIs constitutes a global epidemiological emergency characterised by marked regional heterogeneity in carbapenemase distribution, high attributable mortality and rapidly evolving molecular profiles. Locally adapted surveillance, rapid molecular diagnostics, and stewardship programmes are required since empirical therapy cannot be standardised across regions.

## 1. Introduction

Healthcare-associated infections (HAIs)—defined as infections occurring in patients during the process of care in a hospital or other healthcare facility that were not present or incubating at the time of admission—represent one of the most significant and costly complications of modern medical practice. According to the third point prevalence survey coordinated by the European Centre for Disease Prevention and Control (ECDC), an estimated 4.3 million patients in EU/EEA acute care hospitals acquire at least one HAI annually, corresponding to a prevalence of 7.1% of hospitalised patients [1]. This burden translates into an estimated 2.6 million new cases and approximately 2.5 million disability-adjusted life years per year—a cumulative burden of 501 DALYs per 100,000 general population, exceeding that of influenza, tuberculosis and all other communicable diseases under ECDC surveillance combined; healthcare-associated pneumonia and primary bloodstream infection together account for more than 60% of this burden [2]. Critically, HAIs account for 71% of all infections caused by antibiotic-resistant bacteria, and up to 50% are considered preventable [3].

Among the diverse aetiological agents of HAIs, Gram-negative bacteria—*Klebsiella pneumoniae* in particular—have assumed increasing clinical prominence over the past two decades. A member of the ESKAPE group of pathogens (E—*Enterococcus faecium*, S—*Staphylococcus aureus*, K—*Klebsiella pneumoniae*, A—*Acinetobacter baumannii*, P—*Pseudomonas aeruginosa*, E—*Enterobacter* spp.) [4], *K. pneumoniae* is an opportunistic encapsulated bacillus capable of causing a spectrum of nosocomial infections, including pneumonia, bloodstream infections (BSI), urinary tract infections (UTI), surgical site infections, and meningitis [5].

The global burden attributable to this pathogen is substantial and disproportionately driven by resistance. In 2019, *K. pneumoniae* infections accounted for approximately 800,000 deaths worldwide, of which around 80% were associated with antimicrobial resistance; lower respiratory tract, bloodstream, and intra-abdominal infections together accounted for nearly 90% of these deaths, with mortality driven predominantly by resistance to carbapenems and third-generation cephalosporins [6]. Successive Global Burden of Disease analyses of antimicrobial resistance place *K. pneumoniae* among the small group of pathogens responsible for the greatest share of resistance-associated mortality worldwide, and project a continued rise in that burden to 2050 [7]. This is reflected in current prioritisation frameworks: in the WHO Bacterial Priority Pathogens List 2024, carbapenem-resistant *K. pneumoniae* was the top-ranked pathogen overall, and carbapenem-resistant and third-generation cephalosporin-resistant Enterobacterales were assigned to the critical priority tier [8].

At the European level, *K. pneumoniae* is the pathogen driving the deterioration in the antimicrobial resistance landscape. The estimated EU incidence of carbapenem-resistant *K. pneumoniae* bloodstream infections reached 3.51 per 100,000 population in 2024 (country range 0.02–20.31), 61.0% higher than in the 2019 baseline year and well above the 2030 target of 2.07 per 100,000, with a statistically significant increasing trend [9]. Resistance levels remain highest in southern, central and eastern Europe, where more than 35,000 deaths per year are attributed to infections with resistant bacteria across the EU/EEA [9]. Consistent with this, 32% of microorganisms recovered from microbiologically documented HAIs in the 2022–2023 ECDC point prevalence survey were antimicrobial-resistant [1].

The clinical threat posed by *K. pneumoniae* has been dramatically amplified by the acquisition and horizontal transmission of mobile genetic elements encoding beta-lactamase enzymes. Extended-spectrum beta-lactamases (ESBLs)—primarily CTX-M enzymes—confer resistance to third-generation cephalosporins, while carbapenemases (notably KPC, NDM, OXA-48, VIM and IMP) render organisms resistant to last-resort carbapenem antibiotics [10]. These resistance traits, combined with co-transfer of genes encoding resistance to aminoglycosides, fluoroquinolones and colistin, have produced extensively drug-resistant and pandrug-resistant phenotypes for which therapeutic options are critically limited [11,12,13].

The epidemiological landscape has been further complicated by the emergence and global dissemination of hypervirulent *K. pneumoniae* (hvKP) strains into hospital environments [14]. More alarming still is the convergence of hypervirulence with carbapenem resistance into so-called CR-hvKP strains, which combine the immune evasion properties of hvKP with the near-untreatable resistance profile of CRKP. This dual-threat phenotype, predominantly associated with the ST11-K64 and ST11-KL47 clonal lineages, has been documented across multiple continents and is increasingly reported in intensive care settings [15,16].

The risk profile for CRKP acquisition is well characterised and largely modifiable. In a systematic review that compared CRKP cases against two different control groups, eight factors were associated with infection irrespective of the comparator chosen: admission to an intensive care unit, central venous catheter use, mechanical ventilation, tracheostomy, urinary catheter use, prior antibiotic use, and exposure to carbapenems and aminoglycosides. Prolonged length of hospital stay carried the single largest effect (OR 15.28), while prior hospitalisation, renal dysfunction, neurological disorders, nasogastric tube use, dialysis, and exposure to quinolones, fluoroquinolones, glycopeptides, and vancomycin were identified against one comparator only [17]. Earlier meta-analyses reported a broadly concordant profile [18,19]. The dependence of several estimates on the choice of control group is itself informative and explains part of the inconsistency between published risk-factor analyses [17].

These considerations are particularly relevant for Romania. According to the 2024 ECDC Surveillance Atlas of Infectious Diseases, Romania ranked third in Europe for carbapenem resistance among invasive *K. pneumoniae* isolates, with a resistance rate of 50.30%, surpassed only by Greece (60.20%) and Bulgaria (67.60%) [12,20]. Single-centre longitudinal data illustrate the trajectory and its therapeutic consequences: in a Romanian tertiary infectious diseases hospital, CRKP prevalence rose from 4.9% in 2010 to 41.6% in 2024, accompanied by an overall increase in antibiotic use—including a 186% rise in colistin consumption between 2019 and 2024—while susceptibility of NDM plus OXA-48-like co-producing isolates was only 16.5% to colistin and 58.5% to cefiderocol [21]. The predominance of double carbapenemase producers distinguishes Romanian and other Eastern European isolates from Western European ones and further narrows therapeutic options [12,13]. This occurs against a background of high national antibiotic consumption [22], at a time when the EU population-weighted mean total consumption of antibacterials for systemic use was 20.3 DDD per 1000 inhabitants per day in 2024—2% above the 2019 baseline and 4.4 DDD above the 2030 target of 15.9 [23].

Against this background, the present narrative review synthesises the evidence published between 2020 and 2025 on the global and regional epidemiology, molecular mechanisms, clinical outcomes and therapeutic management of *K. pneumoniae*. HAIs, with a focus on carbapenem-resistant strains, in order to inform infection control strategies, empirical treatment protocols and research priorities in high-prevalence settings such as Romania.

## 2. Literature Search Strategy

This article is a narrative review. It was not conducted as a systematic review and is therefore not reported according to the PRISMA 2020 statement; no protocol was registered, and no new quantitative synthesis was performed. All pooled estimates presented below are cited from the published meta-analyses and surveillance reports in which they were originally calculated, and are attributed to those sources at the point of use.

PubMed/MEDLINE, Embase, Web of Science Core Collection and the Cochrane Central Register of Controlled Trials were searched for publications appearing between January 2020 and June 2025, restricted to the English language. The search was subsequently updated in July 2026 to incorporate key surveillance reports and meta-analyses published during manuscript preparation. The search combined Medical Subject Headings and free-text terms: (“*Klebsiella pneumoniae*”) AND (“healthcare-associated infection” OR “nosocomial infection” OR “hospital-acquired”) AND (“antimicrobial resistance” OR “carbapenem resistance” OR “ESBL” OR “KPC” OR “NDM” OR “OXA-48” OR “multidrug resistant”). Reference lists of retrieved articles and of relevant systematic reviews were screened for additional sources. Surveillance reports from the World Health Organization, including the Global Antimicrobial Resistance and Use Surveillance System (GLASS) database) and from the ECDC were consulted as grey literature.

Priority was given to systematic reviews and meta-analyses, multicentre cohort studies, national and supranational surveillance reports, and clinical guidelines. Publications were selected narratively by the authors on the basis of relevance to the themes addressed, methodological quality and recency; studies reporting exclusively community-acquired infections were not considered. Selection was not performed in duplicate, no formal risk-of-bias instrument was applied, and no assessment of publication bias or certainty of evidence was undertaken. This process yielded a total of 62 sources—comprising systematic reviews and meta-analyses, multicentre cohort studies, national and supranational surveillance reports, and clinical guidelines—which were selected for inclusion and are cited throughout this review. Sources addressing the One Health dimension of CRKP—environmental reservoirs, hospital wastewater and wastewater-based surveillance—were identified through a supplementary targeted search of the same databases performed in July 2026, combining (“*Klebsiella pneumoniae*” OR “carbapenem-resistant *Enterobacterales*”) AND (“wastewater” OR “hospital environment” OR “sink” OR “drain” OR “One Health” OR “environmental surveillance”).

A methodological caveat applies throughout this review and should be borne in mind when interpreting the pooled figures presented in the following sections. Several of the quantitative conclusions emphasised here—global and regional CRKP prevalence (Section 3.1), pooled mortality (Section 5.1) and risk-factor associations (Section 5.2)—rest predominantly on a single large meta-analysis each (Lin et al. [24], Xu et al. [25] and Jin et al. [11], respectively), rather than on converging estimates from multiple independent syntheses; where more than one synthesis exists, discordant findings are noted at the relevant point in the text. These source meta-analyses report extremely high statistical heterogeneity (I^2^ frequently exceeding 95%), reflecting the pooling of primary studies that differ in surveillance case definitions (e.g., point-prevalence versus continuous surveillance, and varying definitions of hospital-acquired infection), in the laboratory methods used to identify carbapenem resistance and carbapenemase production (phenotypic susceptibility testing versus molecular or immunochromatographic detection), and in the carbapenem susceptibility breakpoints applied, which were revised by CLSI and EUCAST during the period spanned by the included primary studies and can shift a given isolate’s resistance classification. Readers should therefore treat the prevalence, mortality and risk-factor figures reported below as indicative of overall burden rather than as precisely comparable, generalisable point estimates; the full set of methodological limitations is discussed in Section 8.2.

## 3. Global and Regional Epidemiology of CRKP in Healthcare-Associated Infections

### 3.1. Global Burden

The most comprehensive quantitative estimate currently available is the systematic review and meta-analysis by Lin et al. [24], which pooled 61 studies comprising 513,307 patients with hospital-acquired *K. pneumoniae* infection from 14 countries and territories, published between 2008 and 2023. In that analysis, the global pooled prevalence of CRKP among nosocomial *K. pneumoniae* infections was 28.69% (95% CI: 26.53–30.86%), with very high statistical heterogeneity (I^2^ = 99.8%). Regional estimates from the same source are summarised in Table 1.

Prevalence refers to the proportion of hospital-acquired *K. pneumoniae* infection episodes—not unique patients or individual isolates—caused by carbapenem-resistant strains, as defined in the source meta-analysis [24]; whether repeat isolates from the same patient were excluded is not reported in the original studies.

At country level, the same analysis reported the highest prevalence in Greece (70.61%; 95% CI: 56.77–84.45%), followed by India (67.62%; 95% CI: 53.74–81.79%) and Taiwan (67.54%; 95% CI: 58.65–76.14%), while Germany, the United States, Tunisia and mainland China each reported rates below 20% [24]. Prevalence was substantially higher in countries with a low or middle socio-demographic index (29.77%) than in high-index countries (18.51%), and reached 62.31% in the intensive care subgroup, although the latter estimate derived from only two study populations and carries a correspondingly wide confidence interval [24].

This nearly five-fold gradient across GBD regions is unlikely to reflect true underlying differences in pathogen biology alone. Several structural factors plausibly interact to produce it. Antimicrobial consumption is a primary candidate: carbapenem and broad-spectrum antibiotic use—a principal selective pressure for carbapenemase acquisition—varies severalfold across the regions represented in Table 1, and is generally higher in health systems with weaker antimicrobial stewardship oversight [9,23]. Infection prevention and control capacity is a second driver: the proportion of hospitals with dedicated IPC teams, single-room isolation capacity and hand-hygiene compliance differs substantially between high-income and low- and middle-income settings, directly affecting the secondary spread of resistant clones once introduced [1]. Third, surveillance intensity and diagnostic capacity are themselves uneven: countries with well-resourced clinical microbiology laboratories and systematic carbapenemase-detection algorithms are more likely to identify and report CRKP accurately, whereas under-resourced settings may under-ascertain true prevalence, biassing the pooled estimates in either direction. Finally, the very high statistical heterogeneity (I^2^ = 99.8%) reported by Lin et al. [24] is itself informative: it indicates that the included studies differ not only in true prevalence but in case-mix, in the definition of hospital-acquired infection applied, and in laboratory methodology, so the pooled figure should be read as an indication of overall burden rather than a directly comparable cross-country benchmark.

Two temporal observations frame these figures. Among the studies included in this review, single-centre Chinese reports documented measurable changes in *K. pneumoniae* resistance patterns across the pre-pandemic, pandemic and post-pandemic periods. [26]. Over a longer horizon, the aetiology of HAI has moved from Gram-positive predominance towards multidrug-resistant Gram-negative organisms and emerging fungal pathogens. Carbapenem resistance in *K. pneumoniae* and *Acinetobacter baumannii* has increased, while methicillin-resistant *Staphylococcus aureus* (MRSA) and *Clostridioides difficile* rates have fallen. Low- and middle-income countries carry a disproportionate share of the Gram-negative burden [27].

### 3.2. Eastern Versus Western Europe, with a Focus on Romania

European surveillance data complement this picture. The ECDC reports that the estimated EU incidence of carbapenem-resistant *K. pneumoniae* bloodstream infections rose by 61.0% between 2019 and 2024 [9], with a steep east–west gradient. Romania is among the most affected countries, at 50.30% carbapenem resistance among invasive isolates, behind Bulgaria (67.60%) and Greece (60.20%) [12,20]. Institutional Romanian series document the same trajectory locally, with CRKP prevalence rising from 4.9% in 2010 to 41.6% in 2024 in one tertiary infectious diseases hospital [21]; national figures reproduced in the Romanian literature indicate a rise in CRKP from 0% in 2007 to 47.8% in 2022 [13].

Several converging factors help explain why Romania and other Eastern European countries occupy the upper end of this gradient. Antimicrobial consumption is disproportionately high: EU-wide surveillance places total antibacterial use at a population-weighted mean of 20.3 DDD per 1000 inhabitants per day in 2024, but national consumption in several Eastern European countries substantially exceeds this mean, and hospital antimicrobial stewardship programmes remain less consistently implemented than in Western Europe [22,23]. Healthcare infrastructure and infection-control resourcing constitute a second axis of difference: ICU bed availability, nurse-to-patient ratios and access to single-room isolation are generally lower in Eastern European hospitals, favouring secondary transmission of resistant clones once introduced [1]. Third, molecular and genomic surveillance capacity has historically been more limited in Eastern Europe, which both delays outbreak containment and may partly explain why the region’s dual-carbapenemase epidemiology (Section 4.2) was characterised only recently [12,13,21,28]. These factors are difficult to disentangle from cross-border patient referral and historical links with high-NDM-prevalence regions of the Indian subcontinent, and their relative contributions cannot be quantified from the available literature.

## 4. Resistance Mechanisms and Molecular Epidemiology

### 4.1. Global Carbapenemase Distribution

Carbapenem resistance in *K. pneumoniae* is driven predominantly by the acquisition of carbapenemase genes carried on mobile genetic elements, with KPC-type serine carbapenemases, NDM-type metallo-beta-lactamases and OXA-48-like oxacillinases accounting for the large majority of isolates worldwide; VIM and IMP enzymes represent minority genotypes [10,12]. The relative distribution of these enzymes is strongly geographically patterned rather than uniform, which is the single most consequential observation for empirical therapy. KPC predominates in the Americas, Italy, Greece and Israel; NDM is characteristic of the Indian subcontinent and increasingly of Eastern Europe; and OXA-48-like enzymes dominate in Turkey, North Africa and the Middle East [10,28].

This regional clustering is best explained by a combination of founder effects and selection pressure rather than by chance. Each carbapenemase family disseminated initially through a small number of successful plasmid–clone combinations that established themselves locally and then expanded clonally within health systems with sufficient patient turnover and cross-transmission opportunity; once established, continued high consumption of the corresponding first-line agents provided sustained selective pressure that entrenched the locally dominant enzyme rather than permitting displacement by imported strains [5,10]. Cross-border patient movement, including medical tourism and repatriation of critically ill patients, is repeatedly invoked as the mechanism by which NDM enzymes—originally described on the Indian subcontinent—became established in Eastern Europe and the Middle East, although the precision of this reconstruction is limited by uneven genomic surveillance coverage across regions [10,28].

### 4.2. Eastern Versus Western Europe: Molecular Contrasts

The most detailed European evidence comes from the EURECA genomic survey, which characterised 687 carbapenem-resistant isolates from 41 hospitals in nine Southern European countries between 2016 and 2018 and compared them with the earlier EuSCAPE collection. *bla_KPC-like_* was the most prevalent carbapenemase gene, present in 46% of isolates, followed by *bla*_*OXA-*48_ in 39%, and eleven major clonal lineages were identified, most isolates belonging to the high-risk clones ST258/512, ST101, ST11 and ST307. Crucially, the pairing of gene and lineage was region-specific: *bla*_KPC-like_ ST258/512 in Greece, Italy and Spain; *bla*_OXA-48_ ST101 in Serbia and Romania; *bla*_NDM_ ST11 in Greece; and *bla*_OXA-48-like_ ST14 in Türkiye [28].

Eastern European isolates are distinguished by the frequent co-production of two carbapenemases, most often NDM together with an OXA-48-like enzyme. This pattern has been documented repeatedly in Romanian tertiary hospitals [12,13,21] and in Ukraine, and contrasts with Western European isolates, in which single-enzyme production predominates [12,28]. Dual carbapenemase production is therapeutically decisive, because it simultaneously removes both ceftazidime-avibactam (inactive against metallo-beta-lactamases) and aztreonam monotherapy (hydrolysed by the co-produced serine enzymes) from the empirical armamentarium. In Greece, by contrast, KPC remains the most commonly encountered carbapenemase [12,29].

### 4.3. Beta-Lactamase and Colistin Resistance Determinants

ESBL production, predominantly involving CTX-M-15 and CTX-M-27 enzymes, is highly prevalent among hospital-acquired *K. pneumoniae* and frequently coexists with carbapenemase carriage and with plasmid-mediated AmpC enzymes [10,24]. Colistin resistance, including plasmid-mediated *mcr* determinants, has been reported in CRKP populations and is of particular concern where colistin remains a mainstay of therapy [10,11,21].

The relative weight of these colistin resistance mechanisms deserves clarification, because plasmid-mediated *mcr* determinants—although epidemiologically important as a horizontally transferable trait—are not the principal route to colistin resistance in *K. pneumoniae*. Chromosomal mechanisms predominate, and the single most frequent event is inactivation of *mgrB*, which encodes a small transmembrane protein exerting negative feedback on the PhoP/PhoQ two-component regulatory system. Loss of MgrB function—most often through insertion-sequence disruption, point mutation, or deletion of the gene—leaves PhoPQ constitutively active, upregulating the lipid A modification pathway and reducing the net negative charge of the outer membrane, so that polymyxin binding is impaired [30]. Regional data are consistent with this: across Balkan countries, colistin resistance among CRKP has been attributed principally to alterations of the chromosomal *mgrB* gene rather than to *mcr* carriage [31]. The distinction is operationally important. Screening for *mcr* alone will substantially under-detect colistin resistance; and because *mgrB*-mediated resistance arises de novo under colistin exposure rather than being acquired horizontally, it constitutes a direct argument for restricting colistin use in settings such as Romania, where consumption has risen steeply (Section 6.2). The mechanism may also carry transmission consequences: in a murine model, an *mgrB*-deficient mutant colonised the gut less efficiently than its parent strain but survived better outside the host and transmitted more readily between animals [30].

The clinical significance of these accompanying determinants is greater than their frequent relegation to a secondary role suggests. Carbapenemase-producing isolates rarely carry the carbapenemase gene alone: co-production of an ESBL—most often CTX-M-15—or of a plasmid-mediated AmpC enzyme is the rule rather than the exception, and it is this combination, rather than the carbapenemase itself, that determines whether an isolate expresses a fully multidrug-resistant phenotype. In a Croatian intensive care series, every OXA-48-producing isolate but one co-harboured a CTX-M-family ESBL [32]. Conversely, French national data show that *bla*_OXA-48_ was the only acquired resistance gene present in a substantial subset of carbapenemase producers, which therefore remained susceptible to most non-carbapenem agents [33]. The corollary is that the resistance phenotype of a carbapenemase producer cannot be predicted from the carbapenemase gene in isolation, a point developed further in Section 4.6.

### 4.4. Global Clonal Lineage Distribution

Molecular typing consistently identifies a limited number of high-risk clonal lineages responsible for the majority of CRKP isolates globally. ST258 and its derivative ST512, both predominantly KPC-carrying, have driven nosocomial outbreaks in Southern Europe, Israel, and the Americas [12,13,29]. ST11 is the dominant clone in China, frequently carrying *bla*_KPC-2_ and increasingly associated with the acquisition of virulence plasmids that generate the CR-hvKP phenotype [34,35]; in one Chinese tertiary centre, all *bla*_KPC-2-positive_ isolates belonged to ST11, with predominant intra-hospital transmission of the ST11-KL64 subclone, whereas *bla*_NDM-5-positive_ isolates were distributed across five distinct sequence types and showed evidence of both clonal and horizontal transmission [34]. ST101, predominantly associated with *bla*_OXA-48-like_, is prevalent in the Balkans, including Romania and Serbia, and has been linked to colistin resistance [12,28]. ST307 and ST147 have expanded across multiple continents, the latter carrying *bla*_NDM_ and *bla*_OXA-181_ and being implicated in emerging ceftazidime-avibactam resistance [10,24,28]. Table 2 summarises the principal lineages and their carbapenemase associations.

The plasmid backbones, resistance determinants and virulence markers associated with these clonal lineages are summarised in Table 3.

Carbapenemase distribution across South-Eastern Europe is summarised in Table 4. Two features deserve emphasis. First, KPC-2 and NDM-1 are the most frequently reported enzymes in Croatia, Bulgaria, Serbia and Slovenia [31], so the dual-carbapenemase pattern described above for Romania is not uniform across the region. Second, the literature is not fully concordant for Romania itself: regional reviews describe KPC as historically dominant since 2007 [31], whereas contemporary Romanian institutional series document a predominance of NDM plus OXA-48-like co-producers [12,13,21]—a discrepancy best interpreted as a genuine temporal shift rather than a contradiction, and one that underlines why carbapenemase typing must be current rather than inferred from historical national profiles. A further regional trend is the apparent replacement of ST258 by ST11 across the Balkan Peninsula since 2018, with ST15 persisting throughout [31].

The concentration of dual-carbapenemase producers in Eastern Europe rather than a uniform pattern across the continent is unlikely to be explained by molecular epidemiology alone. It plausibly reflects the compounding of three factors: sustained high consumption of both carbapenems and broad-spectrum beta-lactams, which co-selects for NDM and OXA-48-like determinants simultaneously; comparatively later adoption of rapid carbapenemase-typing diagnostics in the region, which delays the targeted infection-control response needed to contain emerging co-producing clones before they become endemic; and cross-border transmission routes linking Eastern Europe with high-NDM-prevalence regions, superimposed on an existing OXA-48-like reservoir already present locally [10,12,13,21,28]. The EURECA survey’s finding that gene–lineage pairings are strongly region-specific [28] is itself consistent with locally constrained transmission networks rather than a single pan-European epidemic clone.

### 4.5. Mobile Genetic Elements and the Role of Conjugative Plasmids

The efficiency with which carbapenemase genes disseminate depends heavily on the plasmid backbone carrying them, and this is as important as clonal expansion in explaining the regional patterns described above. The global spread of *bla*_OXA-48_ is attributable less to the success of any single clone than to a single self-transferable IncL/M-type plasmid of approximately 62 kb which, notably, carries no additional resistance gene [42]; its high conjugation frequency allows *bla*_OXA-48_ to move readily between species and between clonal backgrounds, and accounts for its recovery from genetically unrelated isolates within the same institution [33]. Comparative genomic analysis has shown that this plasmid family is not uniform: several pOXA-48 variants retain a complete conjugation system, whereas the pOXA-181 and pOXA-232 derivatives lack one, which plausibly contributes to their more restricted distribution [43]. Broad-host-range IncA/C plasmids play an analogous role for other beta-lactamases, typically assembling large multiresistance regions that combine expanded-spectrum cephalosporinases such as the plasmid-mediated AmpC enzyme FOX-5 with additional antimicrobial and heavy-metal resistance determinants, and have been shown to be acquired by patients during intensive care admission [44]. For *bla*_NDM_, dissemination is distributed across several backbones—IncFII, IncX3, IncFIB, IncL/M and IncA/C have all been reported—with IncX3 plasmids in particular recovered from phylogenetically diverse organisms in both clinical and environmental samples and transferring efficiently at ambient temperature, a property directly relevant to the environmental persistence discussed in Section 7 [45]. For Eastern European settings in which NDM and OXA-48-like enzymes co-circulate, the practical consequence is that infection control cannot be organised around clone tracking alone: plasmid-level surveillance is required to explain why resistance appears simultaneously in unrelated lineages.

### 4.6. Carbapenemase Type, Resistance Phenotype and Implications for Laboratory Detection

The carbapenemase genotypes described above do not translate into a single resistance phenotype, and the differences are large enough to affect both treatment and laboratory detection. KPC-type serine carbapenemases essentially hydrolyse the whole beta-lactam class, including expanded-spectrum cephalosporins and carbapenems, so KPC producers typically present as uniformly and highly carbapenem-resistant and are readily recognised as multidrug-resistant on routine susceptibility testing [5]. OXA-48-like enzymes behave very differently: they hydrolyse penicillins efficiently but carbapenems only weakly, and they spare broad-spectrum cephalosporins almost entirely [42]. An isolate producing OXA-48 without any additional beta-lactamase may therefore show reduced susceptibility to ertapenem while remaining within the susceptible range for imipenem and meropenem, and may retain full susceptibility to third-generation cephalosporins—so that it does not satisfy conventional multidrug-resistance definitions and can pass unremarked in a laboratory relying on phenotypic screening alone [33,42]. High-level carbapenem resistance in OXA-48 producers generally emerges only when enzyme production is combined with porin loss or with co-production of an ESBL or AmpC enzyme [42].

Two practical consequences follow, both bearing directly on the Romanian setting. First, phenotype-based screening will systematically under-ascertain OXA-48-like producers, biassing surveillance towards KPC and metallo-beta-lactamase producers and understating the true reservoir of transmissible carbapenemase genes; because the pOXA-48 plasmid is highly conjugative and may confer no other resistance [33,42], such isolates remain phenotypically inconspicuous while being epidemiologically active. Second, where NDM and OXA-48-like enzymes are co-produced, as predominates in Romanian tertiary hospitals [12,13,21], the phenotype reverts to uniform high-level beta-lactam resistance, and the diagnostic difficulty disappears—but the therapeutic options collapse simultaneously (Section 6.1). The practical implication is that carbapenemase typing should not be reserved for isolates that already appear extensively resistant: it is precisely the phenotypically unremarkable, ertapenem-non-susceptible isolate that most warrants molecular characterisation.

This is the context in which rapid carbapenemase identification, rather than carbapenemase detection alone, becomes therapeutically decisive. Lateral-flow immunochromatographic assays now allow the five most prevalent carbapenemase families—KPC, NDM, VIM, IMP and OXA-48-like—to be identified directly from a bacterial colony within approximately fifteen minutes and without specialised equipment. In a challenge set of 299 molecularly characterised carbapenem-resistant Enterobacterales isolates, one such assay identified all KPC-, NDM-, VIM- and OXA-48-producing isolates, with an overall sensitivity of 100% and specificity of 99.9% [46]. The clinical value of this information lies in its timing: it is available well before phenotypic susceptibility results, and it is immediately actionable. A result indicating a metallo-beta-lactamase excludes the entire class of serine-beta-lactamase inhibitor combinations—ceftazidime-avibactam, meropenem-vaborbactam and imipenem-relebactam are all inactive against these enzymes—and redirects empirical therapy towards aztreonam-avibactam, an aztreonam-containing combination, or cefiderocol (Section 6.1). Conversely, identification of KPC alone supports early use of a serine-inhibitor combination in preference to polymyxin-based therapy. This coupling of rapid genotypic identification to a pre-agreed therapeutic algorithm is the core of diagnostic stewardship as applied to carbapenem-resistant infection [47], and it is of particular value in settings such as Romania, where dual NDM plus OXA-48-like production predominates and where empirical choices made on phenotype alone are likely to be wrong.

The apparent geographic restriction of individual lineages in Table 2 should be interpreted cautiously: whole-genome sequencing and typing capacity is unevenly distributed globally, so regions with well-established genomic surveillance programmes are more likely to have their locally circulating clones characterised and published than regions where such infrastructure is only recently being established, including much of Eastern Europe. The recent description of the Romanian OXA-48-like ST101 clone [12,13] is illustrative: it is plausible that this lineage circulated for some years before local sequencing capacity became sufficient to detect and report it, meaning current clonal maps may under-represent the true diversity and history of CRKP lineages in less-surveilled regions.

## 5. Clinical Outcomes

### 5.1. Mortality

Carbapenem resistance is consistently associated with substantially worse survival. In the meta-analysis by Xu et al. [25], pooled mortality was 42.14% among 2462 patients infected with CRKP, compared with 21.16% among patients infected with carbapenem-susceptible *K. pneumoniae*. Mortality varied markedly by infection site and by patient group, as summarised in Table 5. A subsequent meta-analysis of *K. pneumoniae* bacteraemia reported a pooled odds ratio for 30-day mortality of approximately 3.9 for CRKP compared with non-CRKP bacteraemia, with the excess mortality attributable to CRKP bacteraemia increasing over time [48].

No Romania- or Eastern Europe-specific mortality data for CRKP were identified in the literature search; the comparative series below therefore serve as global context rather than regional benchmarks.

The higher pooled mortality reported for Europe relative to North America in Table 5 (50.06% vs. 33.24%) should not be over-interpreted as reflecting more virulent pathogens; case-mix, access to novel targeted agents and diagnostic speed are more plausible drivers. Differential access to ceftazidime-avibactam and cefiderocol—both of which improve survival relative to polymyxin-based regimens (Section 6.1)—varies with health-system reimbursement pathways and drug availability, and may be more constrained in the lower- and middle-income European health systems that contribute disproportionately to European CRKP series. Diagnostic capacity for rapid carbapenemase identification is a further candidate driver, since time to appropriate targeted therapy is a recognised determinant of survival in carbapenem-resistant Enterobacterales infection. Because the pooled European estimate aggregates heterogeneous national contributions, however, this explanation remains inferential rather than directly demonstrated by the cited studies [25].

Contemporary series are broadly consistent with these pooled estimates. A comparative analysis from a tertiary hospital in northern China confirmed significantly worse outcomes in carbapenem-resistant than in carbapenem-susceptible *K. pneumoniae* infection [49], while a nationwide Taiwanese multicentre study of CRKP bacteriuria illustrates the substantially more favourable prognosis of urinary tract involvement and the limited impact of antimicrobial therapy in that setting [50].

The additional mortality burden attributable to the convergence of carbapenem resistance with hypervirulence remains uncertain. In the meta-analysis by Chen et al. [15], which included 10 studies and 770 patients (224 with CR-hvKP and 546 with classical CRKP), the pooled odds ratio for mortality associated with CR-hvKP was 2.05 (95% CI: 0.89–4.75)—a trend towards higher mortality that did not reach statistical significance. Notably, the effect differed substantially according to how hypervirulence was defined, with a significant association in studies using phenotypic string tests (OR 4.16) but not in those using genotypic definitions (OR 1.05). Since most of the available evidence originates from East Asia, the global generalisability of these findings is limited, and the clinical impact of CR-hvKP should be regarded as biologically plausible but not yet quantitatively established.

### 5.2. Risk Factors for CRKP Acquisition

The most recent and largest synthesis is the meta-analysis by Jin et al. [11], which pooled 51 case–control and cohort studies comprising 13,860 patients (4711 with CRKP infection and 9149 with carbapenem-susceptible infection) published between 1991 and 2024, and examined 43 candidate risk factors, of which 31 were significantly associated with hospital-acquired CRKP infection. The strongest associations were found for antibiotic exposures—tigecycline (OR 5.97; 95% CI: 3.80–9.38), carbapenems (OR 4.79; 95% CI: 3.72–6.18), polymyxins (OR 4.25; 95% CI: 2.02–8.95) and glycopeptides (OR 3.40; 95% CI: 2.65–4.35)—and for the medical environment, notably intensive care admission (OR 4.27; 95% CI: 3.22–5.66) and a longer pre-infection hospital stay (mean difference 14.98 days). Among invasive procedures, bronchoscopy (OR 4.08; 95% CI: 1.40–11.92), tracheal cannulation (OR 3.72; 95% CI: 2.10–6.60), mechanical ventilation (OR 3.61; 95% CI: 2.72–4.78), central venous catheterisation (OR 3.39; 95% CI: 2.40–4.79) and dialysis (OR 3.00; 95% CI: 2.35–3.83) carried the highest risk; endoscopy and surgical drainage were newly characterised as distinct procedural risk factors in that analysis [11].

Two aspects of this synthesis merit emphasis. First, the reported associations should be read as markers of overall illness burden and healthcare exposure rather than as direct causal effects; the authors pooled unadjusted odds ratios because heterogeneous confounder adjustment across the primary studies precluded meaningful pooling of adjusted estimates [11]. Second, and of direct relevance to the present review, subgroup analysis revealed systematic differences between Eastern and Western populations (here, ‘Eastern’ and ‘Western’ refer to the geographic classification used by Jin et al. for Asian versus non-Asian cohorts, not to Eastern versus Western Europe): cephalosporin (OR 2.68 vs. 1.55) and fluoroquinolone exposure (OR 3.58 vs. 1.89) were more strongly associated with CRKP infection in Western populations, whereas invasive procedures and polymyxin exposure carried substantially higher risk in Eastern populations (dialysis OR 4.47 vs. 2.03; mechanical ventilation OR 4.22 vs. 2.34; polymyxin OR 7.11 vs. 3.02) [11]. This suggests that infection prevention priorities are themselves region-specific, favouring procedural control in Eastern settings and antimicrobial stewardship in Western ones. This division of priorities plausibly reflects underlying differences in infection-control resourcing, favouring device-related and procedural risk mitigation, versus antimicrobial stewardship maturity, favouring restriction of high-risk antibiotic classes, rather than a fundamental difference in patient susceptibility between the two groups of countries.

Earlier meta-analyses reported a broadly concordant profile, implicating prolonged hospitalisation, tracheostomy, parenteral nutrition, immunosuppression, and prior carbapenem exposure [17,18,19]. One notable discrepancy concerns underlying conditions: whereas Lin et al. [24] identified haematological malignancy as the strongest single predictor (OR 4.69; 95% CI: 2.80–7.88), Jin et al. [11] found no significant association for haematological malignancy, diabetes or hypertension, and instead identified respiratory disease (OR 2.69), kidney disease (OR 1.47), cardiovascular disease (OR 1.34) and male sex (OR 1.31) as significant. This divergence probably reflects differences in control group definition [17] and in the use of adjusted versus unadjusted estimates, and indicates that comorbidity-based risk stratification for CRKP remains unsettled. The practical implication is nonetheless consistent across syntheses: the dominant determinants—antibiotic exposure, intensive care admission and invasive devices—are modifiable.

### 5.3. Length of Stay and Resource Utilisation

Beyond mortality, CRKP infection imposes a substantial economic burden through prolonged hospitalisation, increased intensive care utilisation, and the cost of salvage antimicrobial regimens. Health-economic modelling is informative in this respect: in a hybrid decision-tree and Markov model of CRKP bloodstream infection conducted from the Chinese healthcare perspective, ceftazidime-avibactam was found to be cost-effective as definitive therapy relative to polymyxin B monotherapy or polymyxin B-based combinations, against a willingness-to-pay threshold of USD 11,600 per quality-adjusted life-year [51]. The higher acquisition cost of newer agents may therefore be offset by reduced mortality and shorter hospitalisation, although such analyses are health-system specific and cannot be transferred directly to European settings. No comparable European or Romanian cost-effectiveness data were identified; this represents a specific evidence gap for the region, addressed further in Section 8.3.

## 6. Antimicrobial Treatment Landscape

### 6.1. Novel Beta-Lactam/Beta-Lactamase Inhibitor Combinations

Ceftazidime-avibactam is active against KPC- and OXA-48-type carbapenemases and is recommended in current IDSA and ESCMID guidance as the preferred agent for infections caused by strains producing these enzymes. Observational and cohort data indicate improved outcomes with ceftazidime-avibactam compared with polymyxin-based salvage regimens, including in high-risk neutropenic patients with KPC-producing Enterobacterales bacteraemia [52] and in a single-centre retrospective series of CRKP infection [53].

Ceftazidime-avibactam has no direct activity against metallo-beta-lactamases. For NDM-producing strains, the combination of aztreonam with ceftazidime-avibactam exploits the stability of aztreonam to metallo-enzymes together with avibactam’s inhibition of co-produced serine carbapenemases and ESBLs, and is the current combination of choice [10,52]. This distinction is directly relevant to Romanian practice, where NDM plus OXA-48-like co-production predominates [12,13,21].

By contrast, in Western European settings where single-carbapenemase producers (typically KPC alone) predominate, ceftazidime-avibactam monotherapy remains frequently sufficient—underscoring why therapeutic protocols cannot be transposed directly from Western to Eastern European settings.

Emergence of resistance to ceftazidime-avibactam, mediated predominantly by KPC mutations such as D179Y or by a shift towards metallo-beta-lactamase production, has been documented following exposure and represents a critical therapeutic vulnerability requiring active surveillance [36]. Cefiderocol offers an additional option, although susceptibility among Romanian NDM plus OXA-48-like co-producing isolates has been reported at only 58.5% [21].

Ceftazidime-avibactam is not the only option against serine carbapenemases. Meropenem-vaborbactam and imipenem-relebactam have both been approved for infections caused by carbapenem-resistant Enterobacterales and are active against KPC producers, alongside ceftolozane-tazobactam for carbapenem-resistant *Pseudomonas aeruginosa* [47]. Neither vaborbactam nor relebactam inhibits metallo-beta-lactamases, and neither reliably restores activity against OXA-48-like enzymes, so their utility in Eastern European settings is largely confined to the KPC-predominant fraction of isolates. Their principal advantage over ceftazidime-avibactam is a different resistance trajectory: because the mutations that compromise ceftazidime-avibactam, such as KPC D179Y, do not necessarily confer cross-resistance, retaining more than one serine-inhibitor combination in the formulary preserves a salvage option. Optimal use of all of these agents nonetheless depends on the laboratory resolving both the presence and the type of carbapenemase and reporting reliable minimum inhibitory concentrations, without which exposure is likely to be either unnecessary or inadequate [47].

For metallo-beta-lactamase producers, the therapeutic position has changed materially during the review period. The improvised combination of aztreonam with ceftazidime-avibactam has now been formalised as a fixed aztreonam-avibactam combination, which in the phase 3 REVISIT trial achieved clinical cure in 68.4% of patients with complicated intra-abdominal infection or hospital-acquired and ventilator-associated pneumonia, compared with 65.7% for meropenem, with 28-day all-cause mortality of 4% versus 7%; the trial was descriptive rather than powered for superiority, and only a minority of baseline isolates were carbapenemase producers, so evidence specific to metallo-beta-lactamase infection remains limited [54]. Cefiderocol, a siderophore cephalosporin that exploits active iron transport to enter the cell and is stable to both serine and metallo-carbapenemases, was compared with best available therapy in the pathogen-focused CREDIBLE-CR trial and showed broadly similar clinical and microbiological efficacy; numerically more deaths occurred in the cefiderocol arm, however, concentrated in the subgroup with *Acinetobacter* infection rather than among the third of patients with *K. pneumoniae* [55]. Taken together with the reported cefiderocol susceptibility of only 58.5% among Romanian NDM plus OXA-48-like co-producing isolates [21], these data indicate that the metallo-beta-lactamase-directed armamentarium, although genuinely expanded, remains narrow and should be protected by carbapenemase-guided prescribing rather than deployed empirically.

These treatment gaps are not purely microbiological. Reimbursement pathways and drug procurement processes for ceftazidime-avibactam, the aztreonam–ceftazidime-avibactam combination and cefiderocol differ across European health systems, and access barriers are generally more pronounced in Eastern European settings with more constrained pharmaceutical budgets, delaying the shift from empirical polymyxin-based therapy to targeted novel-agent regimens even where the latter would be expected to improve survival (Section 5.1). Diagnostic capacity compounds this: selecting the correct combination regimen for a dual-carbapenemase producer requires rapid carbapenemase identification, which is not uniformly available across Romanian and regional laboratories, so empirical therapy frequently precedes definitive molecular characterisation [12,13,21].

### 6.2. Last-Resort and Adjunctive Options

Colistin and polymyxin B remain last-resort options for extensively drug-resistant CRKP infections, particularly where metallo-beta-lactamase producers predominate and newer agents are unavailable [56]. Their utility is constrained by nephrotoxicity, unfavourable pharmacokinetics and rising resistance: in a Romanian series, susceptibility of NDM plus OXA-48-like co-producing isolates to colistin was only 16.5%, against a background of a 186% increase in colistin consumption between 2019 and 2024 [21]. Tigecycline, fosfomycin and trimethoprim-sulfamethoxazole retain partial activity against some CRKP strains but are generally reserved for urinary tract infection or de-escalation because of pharmacodynamic limitations [16].

This trajectory illustrates a self-reinforcing cycle: constrained access to novel beta-lactam/beta-lactamase inhibitor combinations increases reliance on colistin, and the resulting rise in colistin consumption accelerates the erosion of the very last-resort option on which these settings depend.

## 7. One Health Dimensions: Environmental Reservoirs and Dissemination of CRKP

### 7.1. Environmental Reservoirs Within the Hospital

The hospital wet environment is an established reservoir for carbapenemase-producing Enterobacterales, and its role is directly relevant to the persistence of CRKP documented in the preceding sections. In a systematic environmental screening programme in a hospital with endemic KPC-producing organisms, 219 of 475 room sampling events (46%) were positive at a sink drain, sink P-trap, toilet or hopper, with intensive care room type and previous positivity of the site consistently associated with increased risk; whole-genome sequencing confirmed seeding of the environment by colonised patients in at least 6% of room occupations [57]. Drain-associated reservoirs can also transmit in the opposite direction. During a burn-unit outbreak, OXA-48-producing *K. pneumoniae* ST15 was recovered from toilet water in four of six rooms and from drain water shared between rooms, with outbreak isolates isogenic at fewer than 15 alleles, and the strain persisted in two rooms despite two months of daily bleach disinfection [58]. This observation has specific implications for Eastern European settings: because OXA-48-like enzymes form one component of the dual-carbapenemase profile that predominates in Romanian hospitals (Section 4.2), drain-associated persistence is a biologically plausible—and, to date, largely uninvestigated—contributor to the endemicity documented in Romanian tertiary centres [12,13,21].

### 7.2. Dissemination Beyond the Hospital and the Role of Wastewater Surveillance

Hospital effluent connects the nosocomial reservoir to the wider environment. In a two-year study tracking carbapenem-resistant bacteria from patient rooms through the hospital sewer system to the effluent of the receiving wastewater treatment plant, *K. pneumoniae* ST147 was recovered at nearly all sampling sites and all ST147 isolates formed a single core-genome MLST cluster, providing direct genomic evidence that a high-risk clone had disseminated from the hospital into the environment [59]. ST147 is one of the lineages tabulated in Table 2 and is implicated in emerging ceftazidime-avibactam resistance [10,36], so its environmental escape is not epidemiologically trivial.

Conversely, wastewater can be read as a surveillance signal rather than only as a hazard. A Romanian study of municipal wastewater treatment plants with and without hospital sewage input found that carbapenemase gene abundances were distributed evenly across wastewater types—indicating that these genes circulate in the catchment population independently of direct hospital input—and that the resulting gene profiles mirrored clinically reported resistance patterns [60]. In a Spanish wastewater treatment plant, a carbapenemase gene was detected in wastewater several months before the corresponding first clinical case was identified in the associated university hospital, illustrating the early-warning potential of wastewater-based epidemiology [61]. For a country such as Romania, where clinical molecular characterisation capacity is unevenly distributed between centres (Section 3.2 and Section 4.4), wastewater-based genomic surveillance is an attractive complement to isolate-based surveillance, because it samples the whole catchment population rather than only those patients from whom an isolate is cultured, typed and reported.

These observations situate healthcare-associated CRKP within the broader One Health framework, in which carbapenem-resistant organisms are recovered across hospital sewage, wastewater treatment plants, surface waters, sediments, soil and animals, and in which anthropogenic discharge of untreated or partially treated effluent is the principal proposed link [62]. Two caveats temper this interpretation. First, although species-level occurrence across these compartments is well documented, strain-level transmission pathways remain incompletely established, and the direction of transfer is usually inferred rather than demonstrated [62]. Second, no Romanian study identified in this review has tracked CRKP clones from hospital effluent to receiving waters, so the contribution of Romanian healthcare-associated CRKP to environmental dissemination remains unquantified—a specific research gap addressed further in Section 8.3.

## 8. Discussion

### 8.1. Interpretation of the Evidence

Three themes emerge consistently from the evidence reviewed. First, the global burden of carbapenem resistance in healthcare-associated *K. pneumoniae* infection is high but profoundly uneven, and the pooled global figure conceals a nearly five-fold gradient between the least and the most affected regions (Section 3.1); quoting a single global prevalence is therefore of limited practical value. Second, carbapenem resistance is consistently associated with substantially worse survival, with bloodstream infection carrying the worst prognosis (Section 5.1)—although, because the underlying studies are almost entirely observational, this association should not be read as fully attributable mortality. Third, and most consequential for practice, the molecular architecture of resistance differs fundamentally between regions, so that the correct empirical regimen in Greece is not the correct regimen in Romania or in India.

The Romanian situation exemplifies this last point. The national resistance level among invasive isolates and the documented predominance of NDM plus OXA-48-like co-producers (Section 3.2 and Section 4.2) together define precisely the combination against which ceftazidime-avibactam monotherapy fails and for which aztreonam-containing regimens or cefiderocol are required. Because the susceptibility of these co-producing isolates to both cefiderocol and colistin is already limited (Section 6), the therapeutic margin in this population is narrow enough that empirical mis-selection carries a direct survival cost. This argues strongly for routine carbapenemase typing rather than phenotype-only reporting in Romanian hospitals, and makes the diagnostic-capacity gap identified earlier a therapeutic problem rather than merely a surveillance one.

Two areas warrant particular caution in interpretation. The clinical impact of CR-hvKP, although biologically plausible and widely discussed, is not yet established quantitatively: the only dedicated meta-analysis found a non-significant pooled association with mortality and a strong dependence of the effect on the definition of hypervirulence used [15]. Similarly, the evidence supporting newer beta-lactam/beta-lactamase inhibitor combinations derives largely from observational cohorts rather than randomised trials, and is therefore susceptible to confounding by indication.

A third area remains actively contested: the comorbidity profile identifying patients at highest risk of CRKP acquisition differs materially between the two largest syntheses, which disagree on whether haematological malignancy is a dominant predictor or shows no significant association at all (Section 5.2). Because this discrepancy probably reflects differences in control-group definition and in the use of adjusted versus unadjusted estimates rather than a genuine biological difference, comorbidity-based risk stratification for CRKP cannot currently be recommended, and resolving it will require primary studies with pre-specified, harmonised control definitions.

### 8.2. Limitations

This review has limitations, which fall into three groups. The first concerns its methodology. It is a narrative rather than a systematic review: study selection was not performed in duplicate, no formal risk-of-bias instrument was applied, and neither publication bias nor certainty of evidence was assessed. The search was restricted to English-language publications, which is a material constraint in a review centred on South-Eastern Europe, where a proportion of the national surveillance and clinical literature is published in local languages and will therefore have been missed.

The second group concerns the evidence synthesised. Several of the principal quantitative conclusions rest on a single large meta-analysis each, as flagged at the outset (Section 2). Statistical heterogeneity in those analyses was extreme (I^2^ = 99.8% in Lin et al. [24]), reflecting genuine differences in case mix, surveillance intensity, susceptibility testing methodology and carbapenem breakpoint adoption; pooled prevalence figures should therefore be read as an indication of overall burden rather than as generalisable point estimates, and publication bias was formally detected in that analysis (Egger test, *p* = 0.001) [24]. Geographic coverage of the primary literature is uneven, with sparse representation of Africa, Latin America and much of Eastern Europe; notably, the widely cited Western European estimate of 42.05% derives from 13 studies dominated by Italian data and is not representative of Northern Europe. The principal mortality synthesis [25] searched the literature only to December 2015 and predates both the widespread introduction of ceftazidime-avibactam and the post-2020 epidemiological shifts. Almost all underlying studies were observational, so residual confounding by comorbidity, illness severity and appropriateness of empirical therapy cannot be excluded, and reported mortality differences should not be interpreted as fully attributable mortality; the principal risk-factor synthesis pooled unadjusted odds ratios for precisely this reason [11]. Definitions of HAI and the extent of molecular characterisation varied considerably between sources.

The third group concerns material compiled specifically for this review. Table 3 and Table 4 are narrative compilations drawn from heterogeneous primary studies that differ in typing methodology, sampling frame and completeness of reporting; they were not assembled through a systematic protocol and support no quantitative inference. In both tables, an entry of “not reported” indicates that a trait was not assessed or not described in the retrieved sources, not that it is absent, and the country-level distribution in Table 4 reflects reporting intensity and molecular characterisation capacity as much as true epidemiology. Furthermore, the explanations advanced throughout for observed regional differences—antimicrobial consumption, infection prevention resourcing, healthcare infrastructure, surveillance intensity and diagnostic capacity—are ecological and inferential: they are consistent with the data reviewed but were not formally tested against it, and should be regarded as hypothesis-generating rather than demonstrated. Finally, the therapeutic evidence is uneven and time-sensitive, resting partly on descriptive or open-label trials not powered for superiority [54,55], while the regulatory availability and pricing of newer agents differ across European health systems and continue to change, so this part of the review will date more rapidly than the epidemiological sections.

### 8.3. Implications for Practice, Policy and Research

For clinical practice, the marked regional variation in carbapenemase distribution means that empirical therapy for suspected CRKP infection cannot be standardised internationally and must be guided by local molecular epidemiology, supported by rapid carbapenemase detection. For policy, the concentration of risk in modifiable exposures—carbapenem, quinolone and cephalosporin use, intensive care admission, invasive devices—supports investment in antimicrobial stewardship and device-associated infection prevention as the interventions with the greatest expected yield, alongside expansion of molecular surveillance capacity in high-prevalence Eastern European settings. For research, priorities include prospective multicentre cohorts from under-represented regions, standardised outcome and hypervirulence definitions to reduce heterogeneity, systematic surveillance of emerging resistance to ceftazidime-avibactam and cefiderocol, and randomised evidence in patients infected with dual-carbapenemase-producing strains, for whom current recommendations rest on the weakest evidence base.

Looking ahead, the trajectories of Eastern and Western Europe are likely to diverge further: Eastern European settings, including Romania, face an accelerating shift towards dual-carbapenemase production that progressively narrows the therapeutic armamentarium, whereas Western European settings remain more exposed to KPC-driven outbreaks linked to high antimicrobial consumption. These divergent trajectories argue for region-specific, rather than uniform, European stewardship and surveillance targets.

## 9. Conclusions

Carbapenem-resistant *K. pneumoniae* account for approximately one quarter to one third of healthcare-associated *K. pneumoniae* infections worldwide and are associated with roughly a doubling of crude mortality relative to carbapenem-susceptible infection. The burden is concentrated in South Asia, Southern and Eastern Europe and the Middle East, and is driven by regionally distinct carbapenemase profiles, with an accelerating trend towards dual carbapenemase production in Eastern Europe and the convergence of resistance with hypervirulence in East Asia. Romania, with carbapenem resistance exceeding 50% among invasive isolates and a predominance of NDM plus OXA-48-like co-producers, exemplifies the setting in which generic empirical recommendations fail. Locally adapted surveillance incorporating routine carbapenemase typing, rigorous antimicrobial stewardship and the rational deployment of novel beta-lactam/beta-lactamase inhibitor combinations are urgently required to contain this threat.

## Figures and Tables

**Table 1:** Pooled prevalence of carbapenem resistance among hospital-acquired *Klebsiella pneumoniae* infections, by GBD region, as reported by Lin et al. [24].

I^2^ (%)	Participants (n)	Studies (n)	Pooled CRKP Prevalence (95% CI)	Region
87	552	4	66.04% (54.22–77.85)	South Asia
99.6	158,828	13	42.05% (28.05–50.05)	Western Europe
93.3	3543	14	26.60% (20.26–32.93)	North Africa and Middle East
98.8	343,505	24	20.95% (18.99–22.91)	East Asia
90.5	6879	6	14.29% (6.50–22.08)	High-income North America
99.8	513,307	61	28.69% (26.53–30.86)	Global pooled estimate

CI, confidence interval; CRKP, carbapenem-resistant *Klebsiella pneumoniae*; GBD, Global Burden of Disease. All values are reproduced from Lin et al. [24]; no new pooling was performed for the present review. The very high I^2^ values indicate that these estimates should be interpreted as an indication of overall burden rather than as precise generalisable figures.

**Table 2:** Major *Klebsiella pneumoniae* clonal lineages in HAIs: genotypic and geographic associations reported in the literature (2020–2025).

References	Clinical Significance	Geographic Distribution	Dominant Carbapenemase (s)	Sequence Type
[12,13,29]	Historically the most widespread lineage; nosocomial outbreaks	USA, Italy, Greece, Israel	*bla*_KPC_ (KPC-2, KPC-3)	ST258/ST512
[34,35]	Dominant clone in China; associated	China, East Asia	*bla*_KPC-2_, *bla*_NDM-5_; CR-hvKP convergence	ST11
	with the dual-threat resistant and			
	hypervirulent phenotype			
[12,13,29]	Rising prevalence; colistin	Balkans, South-Eastern Europe, Middle East	*bla*_OXA-48-like_, *bla*_NDM_	ST101
	resistance reported			
[10,24,28]	Broad capacity for resistance gene	Global (emerging)	*bla*_NDM_, *bla*_OXA-48-like_, *bla*_KPC_	ST307
	acquisition; expanding globally			
[10,36]	Community spillover; emerging ceftazidime-avibactam resistance	South Asia, Middle East	*bla*_NDM_, *bla*_OXA-181_	ST147
[10,24]	Nosocomial outbreaks	Turkey, Middle East, North Africa	*bla*_OXA-48-like_ (incl. OXA-232), *bla*_NDM_	ST14

CR-hvKP, carbapenem-resistant hypervirulent *K. pneumoniae*. The table summarises lineage–genotype–geography associations reported in the cited studies; it does not represent a pooled quantitative analysis.

**Table 3:** Predominant plasmid backbones, associated resistance determinants, and virulence markers reported for the major carbapenem-resistant *Klebsiella pneumoniae* clonal lineages.

References	Virulence/Hypervirulence Markers	Associated Resistance Determinants	Predominant Plasmid Backbone (s)	Sequence Type
[37]	Not characteristically reported; classical multidrug-resistant lineage	*bla*_KPC-2_, *bla*_KPC-3_; co-resistance to fluoroquinolones and aminoglycosides	IncFII(K) pKpQIL-type; in ST512, IncFII (K2:A–:B–) identical to pKpQIL	ST258/ST512
[31,34,35,38,39]	Yersiniabactin (*ybt*, ICE*Kp*11); virulence-plasmid acquisition generating the CR-hvKP phenotype; KL64 capsule expansion, with hypervirulent CRKP rising from 28.2% to 45.7% (2016–2020)	*bla*_KPC-2_, *bla*_NDM-1_, *bla*_NDM-5_, *bla*_DHA-1_; colistin resistance via *mgrB* alteration	IncFII/pKPX-1 and IncR (carrying *bla*_NDM-1_ within Tn*125*A); IncL (*bla*_OXA-48_)	ST11
[31,32,39]	Not consistently reported	*bla*_OXA-48-like_, *bla*_NDM_; colistin resistance predominantly via *mgrB* alteration rather than *mcr* in the Balkans	IncL (*bla*_OXA-48_)	ST101
[39,40]	Lower virulence than ST11 in comparative assays	*bla*_NDM-5_, *bla*_KPC_, *bla*_OXA-48-like_, *bla*_CTX-M-15_; ceftazidime-avibactam resistance in NDM-5-producing outbreak isolates	IncFII(K) (*bla*_CTX-M-15_); IncX3 (*bla*_NDM-5_, highly conjugative)	ST307
[37]	Not consistently reported in the cited studies	*bla*_NDM_, *bla*_OXA-181_, *bla*_KPC-3_; co-resistance to fluoroquinolones and aminoglycosides	IncFII (K1:A–:B–) carrying *bla*_KPC-3_; genetic environment conserved with pKpQIL	ST147
[10,24]	Not reported	*bla*_OXA-48-like_ (including OXA-232), *bla*_NDM_	Not characterised in the sources reviewed	ST14

CR-hvKP, carbapenem-resistant hypervirulent *K. pneumoniae*; ICE, integrative conjugative element. Entries reflect associations reported in the cited studies and are not exhaustive; "not reported" indicates that the trait was not assessed or not described in the sources retrieved, rather than demonstrated absence.

**Table 4:** Reported dominant carbapenemase types and clonal lineages among carbapenem-resistant *Klebsiella pneumoniae* in South-Eastern European countries.

References	Note	Clonal Lineage (s) Reported	Predominant Carbapenemase Type (s) Reported	Country
[28,29,31]	Ceftazidime-avibactam-resistant CRKP reported mostly from this setting	ST258/512, ST11	KPC dominant since 2007; NDM and VIM also reported; dual KPC/NDM producers described	Greece
[12,13,20,21,28,31]	50.30% carbapenem resistance among invasive isolates	ST101, ST147	NDM plus OXA-48-like co-production in contemporary series; KPC reported as historically dominant	Romania
[20,31,38]	Highest EU rate of carbapenem resistance among invasive isolates (67.60%)	ST11 (NDM-1 lineage related to the Polish clone)	KPC-2 and NDM-1 most frequent; OXA-48 also reported	Bulgaria
[28,31]	—	ST101 (*bla*_OXA-48_)	KPC-2 and NDM-1 most frequent; OXA-48 reported	Serbia
[31,32]	OXA-48 producers co-harboured CTX-M-family ESBLs in all but one isolate	Not specified in the cited reports	KPC-2 and NDM-1 most frequent; OXA-48 carried on an IncL plasmid in intensive care isolates	Croatia
[31]	—	Not specified in the cited reports	KPC-2 and NDM-1 most frequent	Slovenia
[31,41]	CRKP accounted for 20% of *K. pneumoniae* isolates in a Sarajevo tertiary centre	Not reported	OXA-48 reported; carbapenemase types incompletely characterised	Bosnia and Herzegovina
[31]	Few published reports identified	Not reported	OXA-48 reported; data sparse	Montenegro, Albania
[10,28]	—	ST14	OXA-48-like predominant	Türkiye

CRKP, carbapenem-resistant *Klebsiella pneumoniae*; ESBL, extended-spectrum beta-lactamase. Entries summarise carbapenemase types reported in the cited sources; reporting intensity and molecular characterisation capacity differ markedly between countries, so absence of a genotype from this table should not be read as evidence of its absence in that country.

**Table 5:** Pooled mortality among patients infected with carbapenem-resistant *K. pneumoniae*, by infection site, setting and carbapenemase type, as reported by Xu et al. [25].

Comparator/Note	Pooled Mortality (%)	Subgroup
vs. 21.16% for carbapenem-susceptible strains	42.14	All CRKP infections
Highest site-specific mortality	54.3	Bloodstream infection
Lowest site-specific mortality	13.52	Urinary tract infection
—	48.9	Intensive care unit admission
—	43.13	Solid organ transplantation
95% CI: 38.61–56.79	47.66	KPC-producing isolates
95% CI: 35.81–57.73	46.71	VIM-producing isolates
North America 33.24; Asia 44.82	50.06	Europe (all sites)

CI, confidence interval; CRKP, carbapenem-resistant *Klebsiella pneumoniae*. Values are reproduced from Xu et al. [25], whose search extended to December 2015; more recent regional estimates may differ.

## Data Availability

No new data were created or analyzed in this study. Data sharing is not applicable to this article.
